# Application of directly observed procedural skills in hospital infection training: a randomized controlled trial

**DOI:** 10.3389/fmed.2025.1509238

**Published:** 2025-05-13

**Authors:** Zhumin Hu, Weipeng Zhang, Minyan Huang, Xiaoyan Liu

**Affiliations:** The Affiliated Panyu Central Hospital, Guangzhou Medical University, Guangzhou, Guangdong, China

**Keywords:** Direct Observation of Procedural Skills, hospital infection, training, application, satisfaction

## Abstract

**Objective:**

To evaluate the effectiveness of the Direct Observation of Procedural Skills (DOPS) method in enhancing hospital infection training.

**Methods:**

A total of 196 infection control staff from clinical departments were selected from a hospital and divided into a control group and an experimental group. The control group received conventional hospital infection control, which training included 10 h of theory lectures and 5 h of practical demonstrations, while the experimental group received three formative assessments using the DOPS method in addition to the conventional training at two-month intervals. The results of the three DOPS assessments in the experimental group were analyzed and compared. The training effectiveness was further evaluated by comparing theory test scores and satisfaction ratings between the two groups.

**Results:**

Among these 196 study subjects, the majority were over 35 years old, female, doctors or nurses, with middle titles, a bachelor’s degree, and over 10 years of working seniority. The scores of using protective equipment and the handling of emergencies increased over the three DOPS evaluations (3.93 vs. 3.94 vs. 4.15 and 2.37 vs. 2.53 vs. 2.68, respectively). After adjusting for all covariates, the overall theoretical knowledge score in the experimental group was 7.968 times higher than that of the control group. The number of participants in the experimental group who were satisfied with the training was 86 compared to 71 in the control group for knowledge retention, 82 vs. 62 for clinical application, 83 vs. 67 for knowledge extrapolation, 88 vs. 73 for training methods, and 89 vs. 59 for motivation.

**Conclusion:**

Hospital infection control skills are important for clinical procedural skills. This study found that the application of the DOPS method in infection control training improved trainees’ practical skills, knowledge retention, and ability to implement infection control measures effectively in clinical settings. These results highlight the value of DOPS as a targeted intervention to enhance infection control training outcomes, supporting its further promotion in clinical education programs.

## 1 Introduction

Training in hospital infection management is crucial for medical personnel to acquire knowledge on infection prevention and control, master relevant skills, and enhance their capacity for infection prevention and control (1). In China, where the healthcare system is rapidly expanding to meet the needs of a large and aging population, infection control training plays a critical role in maintaining high standards of patient safety and healthcare quality. Through systematic training, medical personnel can gain a comprehensive understanding of the associated risks of hospital infections, transmission pathways, and the latest preventive and control measures, ensuring they can effectively respond to and manage infection risks in clinical practice. Infection control training that adheres to hospital infection control guidelines is not only a necessary path for improving the professional competence of medical personnel but also an effective method for preventing and controlling hospital infections (2). With this targeted training, medical personnel can better ensure patient safety and reduce the occurrence of nosocomial infections. The outbreaks of Severe Acute Respiratory Syndrome in 2003 and the global spread of COVID-19 in 2019 revealed deficiencies in the knowledge and skills of some medical personnel regarding infection prevention and control in hospitals (3). These major health crises exposed the unpreparedness and knowledge gaps in the existing medical system when dealing with sudden infectious outbreaks, further emphasizing the urgency and importance of hospital infection prevention and control training.

China’s unique healthcare challenges, including a high patient-to-healthcare worker ratio, resource disparities between urban and rural areas, and the need for rapid response systems in densely populated regions, further underscore the importance of rigorous and standardized infection prevention training. To improve infection control levels across medical institutions in the country, China’s National Health Commission issued the “Basic System for Infection Prevention and Control in Medical Institutions” in 2019, which explicitly requires that comprehensive infection control training for all staff be incorporated into the basic institutional framework (4). The introduction of this policy marked a shift from voluntary infection control training to a mandatory institutional requirement, promoting standardized management in infection control across medical institutions. Furthermore, all training and educational activities must rely on a scientific system for assessment and evaluation (5). The establishment of a standardized assessment and evaluation system for training not only provides quantitative evidence for the effectiveness of the training but also helps identify deficiencies in the training process, allowing for targeted improvements in teaching content and methods. A well-structured assessment and evaluation system is a crucial tool for enhancing teaching quality, optimizing training outcomes, and ensuring that medical personnel truly grasp the skills for infection prevention and control (1). By establishing and improving the training assessment mechanism, medical institutions can continuously enhance their infection control capabilities, ensuring the effective operation of the hospital infection management system and ultimately improving patient safety and health protection (6).

The Direct Observation of Procedural Skills (DOPS) is a widely used and important assessment and teaching method in standardized residency training (7). The core concept of DOPS is that, within a real clinical practice setting, the supervising instructor directly observes the trainee performing various clinical procedures, providing real-time guidance and evaluation (8–10). Through this assessment approach, the instructor can thoroughly observe the trainee’s procedural skills, clinical reasoning, and responsiveness, and provide detailed feedback after the procedure, helping the trainee identify strengths and weaknesses, thereby facilitating immediate improvement and skill enhancement. Compared to traditional procedural skill assessment methods, DOPS is not merely a one-time evaluation tool; it places greater emphasis on the trainee’s long-term growth and progress, reflecting the characteristics of formative assessment (11). China’s previously introduced policies emphasize mandatory, comprehensive, and scientifically assessed training for healthcare personnel to enhance infection prevention capabilities. However, traditional training methods often lack real-time feedback and fail to emphasize the application of skills in clinical settings. This limitation creates a gap between theoretical knowledge and practical implementation, potentially undermining infection control efforts. DOPS offers a solution by enabling the direct observation and real-time assessment of procedural skills in actual clinical environments. It not only ensures that trainees acquire knowledge but also demonstrates their ability to apply infection control measures, such as aseptic techniques, personal protective equipment (PPE) usage, and environmental decontamination procedures. By incorporating immediate feedback, DOPS addresses the shortcomings of traditional assessment methods and aligns with the policy’s mandate for evidence-based evaluation and continuous improvement in training outcomes.

DOPS is currently more frequently applied in standardized residency training, and there is limited research on integrating DOPS with hospital infection training. Therefore, this study draws on the DOPS methodology and applies it to teaching, formative assessment, and feedback on hospital infection management. The study hypothesizes that integrating DOPS into hospital infection management training will enhance the effectiveness of training programs by improving trainees’ practical infection prevention skills, adherence to infection control guidelines, and overall competency in managing hospital infection risks.

## 2 Materials and methods

### 2.1 Study population

From 2022 to 2023, 196 infection control staff from the clinical departments of Panyu Central Hospital, a tertiary care hospital, were selected as study subjects (Supplementary Figure 1). The ethics of this study was approved by the Medical Ethics Committee of Panyu Central Hospital (PYRC-2023-187). These participants were randomly divided into a control group and an experimental group, with 98 members in each group. The sample size of 196 participants was determined using G*Power software, based on the following statistical assumptions: effect size: a moderate effect size of 0.5 was assumed, given prior studies on the effectiveness of DOPS in improving procedural skills; power (1-*β*): to achieve 80% power, ensuring a high probability of detecting a true difference if one exists; significance level (*α*): a two-tailed significance level of 0.05 was set; allocation ratio: a 1:1 ratio between the control and experimental groups was used. The calculated minimum sample size for each group was 88. To account for potential dropout or noncompliance, an additional 10% was added, resulting in a final target sample size of 98 participants per group. Block randomization was used to ensure balance in department types and baseline characteristics, the randomization process was conducted by an independent researcher who was not involved in the training or assessment process. Group allocation was concealed using sequentially numbered, opaque, sealed envelopes, which were opened only after participant enrollment to maintain allocation concealment. Blinding was implemented at multiple levels to reduce bias. Participants were blinded to their group assignments to prevent performance bias, and assessors conducting formative evaluations were also blinded to the group allocations to eliminate observer bias. Only the researchers involved in the design and oversight of the study had access to the randomization list. To ensure the validity of the results, a statistical comparison of baseline characteristics between the two groups was conducted to confirm equivalence. During data analysis, all baseline characteristics were included as covariates for adjustment. The control group received conventional training on the basics of hospital infection knowledge and theory, covering five main areas: the use of protective equipment, hand hygiene standards, aseptic techniques, prevention of needlestick injuries, and medical waste classification. The conventional training included 10 h of theory lectures and 5 h of practical demonstrations. The experimental group, in addition to receiving the same conventional training, participated in three formative assessments using the DOPS method at two-month intervals. Each assessment was conducted by trained evaluators over a two-hour period.

### 2.2 DOPS effectiveness evaluation

The DOPS evaluation form consists of four parts: basic information, evaluation items, satisfaction, and feedback. The evaluation items mainly include eight aspects: ① Preparation of materials before the procedure: whether the materials are complete and within the valid period; ② Use of protective equipment: correct selection and wearing of protective equipment according to the principles of standard and additional precautions; ③ Aseptic technique: use of disinfectants, disinfection range, and aseptic procedural standards; ④ Hand hygiene of medical staff: choice of hand hygiene opportunities and correctness of hand hygiene methods; ⑤ Prevention of needlestick injuries: environmental and behavioral controls, such as avoiding two-handed recapping of needles; ⑥ Classification of medical waste: classification of five types of medical waste and use of appropriate containers; ⑦ Emergency response to unexpected situations; and ⑧ Overall performance. The evaluation uses a six-level scale: Levels 1–2 indicate that the trainee’s performance in the item does not meet hospital infection control requirements (major issues); Levels 3–4 indicate that the trainee’s performance meets hospital infection control requirements (no major issues, though with some deficiencies in detail); Levels 5–6 indicate that the trainee’s performance is excellent (procedures are standardized, and details are well-executed). Each item is evaluated independently without affecting the others. The DOPS assessments were carried out in a controlled environment to simulate real clinical scenarios, with trained evaluators observing participants as they performed procedural tasks. Evaluators used a standardized “Hospital Infection Procedural Skills DOPS Scoring Form” to record their observations. After each assessment, immediate and structured feedback was provided to the participants. The feedback included highlighting aspects of the procedure where the participants performed well, identifying specific deficiencies, and offering actionable suggestions for improvement. Additionally, participants were encouraged to reflect on their performance and ask questions to clarify their understanding.

### 2.3 Training effect evaluation of the control group and experimental group

After completing the training, both groups underwent a theory test on basic knowledge of hospital infection. A question bank was created based on key points of hospital infection training, and 100 questions were randomly selected from the bank for the test. The test content primarily covered five areas: the use of protective equipment, hand hygiene standards, aseptic techniques, prevention of needlestick injuries, and classification of medical waste, with each area worth 20 points, for a total score of 100 points.

### 2.4 Satisfaction evaluation of hospital infection training

Satisfaction was evaluated using a questionnaire designed with a 5-point Likert scale, where 1 represented “very dissatisfied” and 5 represented “very satisfied.” The evaluation content included understanding and memorization of knowledge, assistance with clinical practice, ability to apply and integrate knowledge, the rationality of the training methods, and the motivation and initiative of the trainees. Each aspect was assessed through specific questions, and participants rated their satisfaction based on their subjective experience. For instance, questions included: “How effective was the training in enhancing your understanding and memorization of key concepts?” and “To what extent did the training help improve your clinical practice?” The questionnaire was distributed to both groups after they completed the theory test, with each infection control staff member completing one questionnaire. A total of 198 questionnaires were distributed and collected, resulting in a 100% response rate. The average scores for each aspect were calculated to reflect overall satisfaction levels and provide insight into the perceived effectiveness of the training program.

### 2.5 Statistical analysis

SPSS 22.0 and R software version 4.2.0 were used for statistical analysis in this study. All data were expressed as percentages for categorical variables. Chi-square tests were used to analyze differences in basic information between the control and experimental groups, with the measure of association being the chi-square statistic to assess the success of randomization. Analysis of variance (ANOVA) was employed to compare differences in DOPS scores across three assessments. The measure of association was the F-statistic, which indicated overall group differences, and *post hoc* tests were applied to identify specific differences between groups. Independent t-tests were used to analyze differences in the theory test scores on hospital infection knowledge between the two groups. Linear regression was applied to further analyze the differences in theory test scores while adjusting for covariates. The measure of association was the regression coefficient (*β*), along with its 95% CI to evaluate the effect of group allocation on scores and quantify the adjusted differences. Subgroup analysis was conducted to assess potential interaction effects between basic characteristics and group allocation on theory assessment scores. Basic characteristics were stratified into meaningful categories (e.g., age: ≤35 years vs. >35 years; gender: male vs. female) to examine how these variables influenced group differences. The rationale for subgroup analysis was to explore whether certain participant characteristics moderated the impact of the intervention, thus contributing to a deeper understanding of the effectiveness of the training program in different demographic or professional contexts. The scores of each theory assessment were divided into two groups according to the median score. Satisfaction was categorized as satisfactory and unsatisfactory based on the median satisfaction score, and the experimental and control groups were analyzed using the chi-square test. All statistical tests were two-sided with a significance level of *α* = 0.05. R language packages such as “compareGroups” “publish” “forestploter” were used for advanced statistical analyses and visualization of results, including the generation of forest plots to illustrate subgroup findings and the effects of the intervention.

### 2.6 Quality control

The number of part-time infection control personnel, their years of work experience, and the fact that all were undertaking infection control work for the first time were kept consistent. The same trainers provided training during the study period. Infection control management instructors involved in DOPS evaluation received homogenized training on the formative evaluation scheme and implementation methods. They observed the four major puncture procedures of infection control doctors and the intravenous catheter operations of nurses and evaluated them in relation to the DOPS scoring form. A double-entry system was used to reduce data input errors, and final data verification was conducted.

## 3 Results

### 3.1 Basic characteristics of the subjects

The basic characteristics of the 196 participants in this study are shown in Table 1. Among these study subjects, the majority are over 35 years old, female, doctors or nurses, with middle titles, a bachelor’s degree, and over 10 years of working seniority, accounting for 57.1, 77.0, 88.7, 57.2, 68.4, and 61.7%, respectively. There were no significant differences between the control group and the experimental group in terms of basic characteristics (all *p* > 0.05), confirming the effectiveness of the randomization process in ensuring baseline equivalence.

**Table 1:** Characteristics of the study population (*n* = 196).

Variables	Total (*n* = 196)	Group		P*
		Control group (*n* = 98)	Experimental group (*n* = 98)	
Age (%)				0.773
≤35 years	84 (42.9)	43 (43.9)	41 (41.8)	
>35 years	112 (57.1)	55 (56.1)	57 (58.2)	
Sex (%)				0.865
Male	45 (23.0)	23 (23.5)	22 (22.4)	
Female	151 (77.0)	75 (76.5)	76 (77.6)	
Careers (%)				0.393
Doctor	83 (42.3)	46 (46.9)	37 (37.8)	
Nurses	91 (46.4)	41 (41.9)	50 (51.0)	
Others	22 (11.3)	11 (11.2)	11 (11.2)	
Title (%)				0.229
Junior	61 (31.1)	25 (25.5)	36 (36.7)	
Middle	112 (57.2)	60 (61.2)	52 (53.1)	
High	23 (11.7)	13 (13.3)	10 (10.2)	
Education (%)				0.655
Lower than bachelor degree	22 (11.2)	13 (13.3)	9 (9.2)	
Bachelor degree	134 (68.4)	65 (66.3)	69 (70.4)	
Higher than bachelor degree	40 (20.4)	20 (20.4)	20 (20.4)	
Working seniority (%)				0.659
≤10 years	75 (38.3)	39 (39.8)	36 (36.7)	
>10 years	121 (61.7)	59 (60.2)	62 (63.3)	

*P for comparison between the control and experimental groups.

### 3.2 Experimental group DOPS scores

The comparison of the three DOPS evaluations in the experimental group is shown in Table 2. The scores for the use of protective equipment and the handling of emergencies slightly increased over the three evaluations. In the pairwise comparisons, we found a significant difference between the first and third evaluations for hand hygiene, with scores of 4.18 ± 0.36 compared to 4.28 ± 0.37. Additionally, for the handling of emergencies, statistically significant differences were observed in all three pairwise comparisons across the DOPS evaluations (all *p* < 0.05). No statistically significant differences were found in other pairwise comparisons (*p* > 0.05).

**Table 2:** The comparison of three times DOPS scores in the experimental group.

Variables	DOPS			F	*P*	Comparison between groups (*P*)		
	1	2	3			1 vs. 2	1 vs. 3	2 vs. 3
Preparation before the operation	3.93 ± 0.68	4.09 ± 0.61	4.11 ± 0.66	2.233	0.109	0.254	0.167	0.989
Use of protective equipment	3.93 ± 0.71	3.94 ± 0.72	4.15 ± 0.68	2.878	0.058	1.00	0.094	0.124
Aseptic techniques	3.8 ± 0.61	3.8 ± 0.6	3.9 ± 0.63	0.86	0.424	1.00	0.602	0.589
Hand hygiene norms	4.18 ± 0.36	4.13 ± 0.37	4.28 ± 0.37	3.819	0.023	0.711	0.216	0.022
Prevention of needlestick injuries	3.72 ± 0.56	3.68 ± 0.59	3.68 ± 0.61	0.102	0.903	0.966	0.977	1.000
Medical waste classification	3.62 ± 0.6	3.58 ± 0.58	3.69 ± 0.54	0.996	0.371	0.961	0.726	0.402
Emergency response to unexpected situations	2.37 ± 0.29	2.53 ± 0.28	2.68 ± 0.36	24.673	<0.001	<0.001	<0.001	0.002
Overall performance	3.67 ± 0.55	3.71 ± 0.56	3.81 ± 0.63	1.539	0.216	0.955	0.265	0.536

### 3.3 Comparison of theoretical knowledge assessment

Table 3 presents the t-test results for the theoretical knowledge scores between the control group and the experimental group. The scores for various theoretical knowledge assessments in the experimental group were generally higher than those in the control group, except for the use of protective equipment (16.06 vs. 16.86, *p* = 0.081). Table 4 shows the results of the multivariate linear regression analysis. After adjusting for all covariates, including age, sex, career, title, education, and working seniority, we found that the overall theoretical knowledge score in the experimental group was 7.968 (95% CI: 4.721, 11.216) times higher than that of the control group.

**Table 3:** The comparison of theoretical knowledge scores of the control and experimental groups.

Variables	Control group (*n* = 98)	Experimental group (*n* = 98)	*t*	*P*
The use of protective equipment	16.06 ± 3.34	16.86 ± 3.01	−1.753	0.081
Hand hygiene norms	15.27 ± 4.40	17.16 ± 3.00	−3.531	0.001
Aseptic techniques	15.00 ± 3.87	17.06 ± 3.06	−4.141	<0.001
Prevention of needlestick injuries	17.45 ± 3.05	19.02 ± 1.53	−4.561	<0.001
Medical waste classification	16.57 ± 2.86	18.39 ± 1.94	−5.206	<0.001
Total point	80.35 ± 13.57	88.49 ± 8.33	−5.062	<0.001

**Table 4:** Results of multifactor linear regression of theoretical knowledge scores of the control and experimental groups.

Variables	Model	Control group	Experimental group
The use of protective equipment	a	Ref	0.796 (−0.100, 1.691)
	b	Ref	0.775 (−0.116, 1.666)
	c	Ref	0.674 (−0.216, 1.565)
Hand hygiene norms	a	Ref	1.898 (0.838, 2.958)
	b	Ref	1.904 (0.838, 2.969)
	c	Ref	1.873 (0.793, 2.954)
Aseptic techniques	a	Ref	2.061 (1.079, 3.043)
	b	Ref	2.048 (1.064, 3.032)
	c	Ref	2.014 (1.005, 3.022)
Prevention of needlestick injuries	a	Ref	1.571 (0.892, 2.251)
	b	Ref	1.552 (0.878, 2.227)
	c	Ref	1.530 (0.846, 2.214)
Medical waste classification	a	Ref	1.816 (1.128, 2.504)
	b	Ref	1.816 (1.125, 2.508)
	c	Ref	1.877 (1.177, 2.577)
Total point	a	Ref	8.143 (4.970, 11.315)
	b	Ref	8.095 (4.916, 11.274)
	c	Ref	7.968 (4.721, 11.216)

Model a was not adjusted for any covariates; Model b was adjusted for age and sex; Model c was further adjusted for careers, title, education, and working seniority.

### 3.4 Subgroup analysis

**Figure 1:**
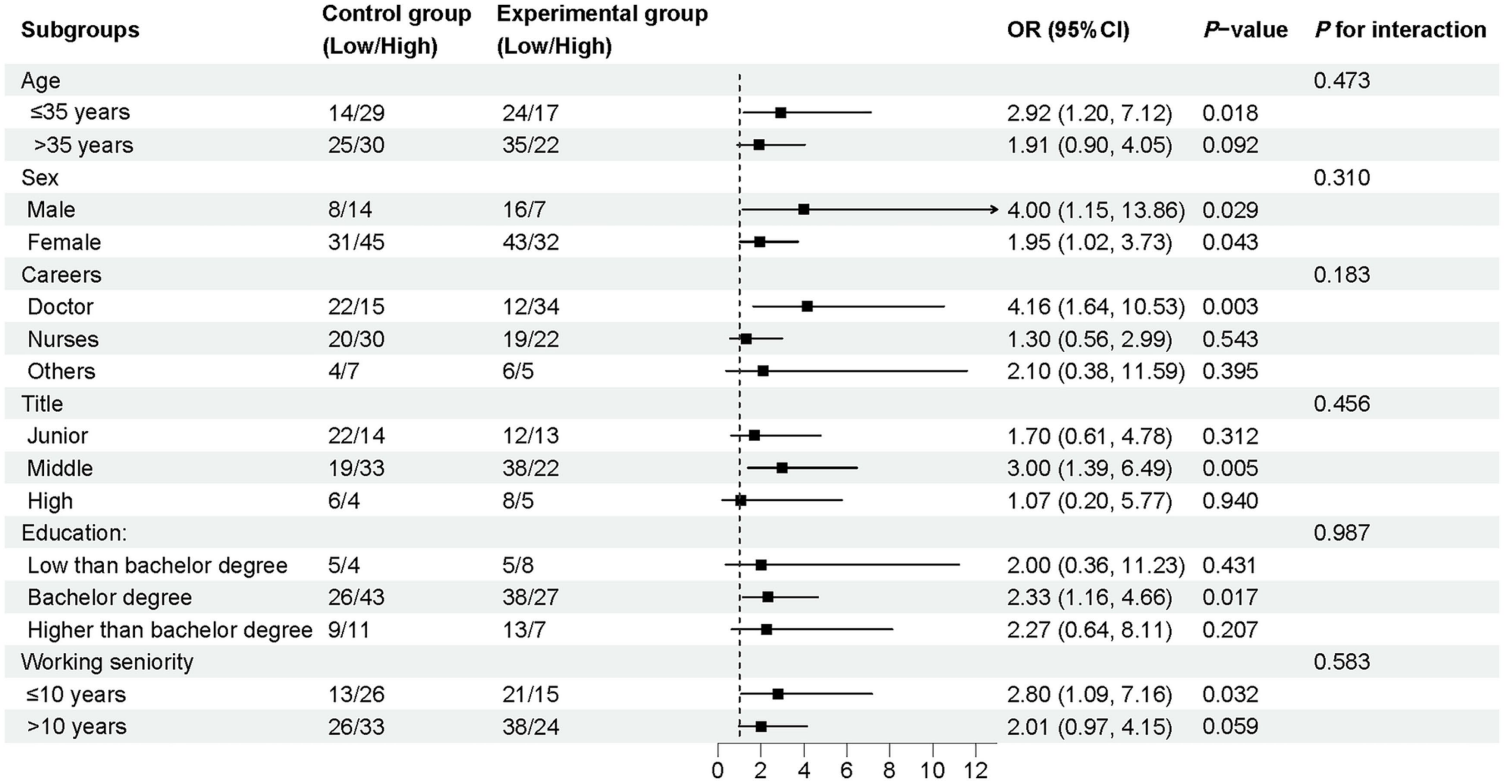
Subgroup analysis of the odds ratio of total point comparing the control and experimental groups.

Figure 1 shows the results of the subgroup analysis of other covariates in relation to the total theoretical knowledge assessment scores for both the control and experimental groups. From the results in Figure 1, none of the covariates exerted an interactive effect on the total score in either the control or experimental group (all *p* > 0.05). Additionally, we conducted subgroup analyses for each theoretical knowledge assessment, as shown in Supplementary Figures 2–6, and similarly, no interactive effects on the individual assessment results were observed.

### 3.5 Satisfaction evaluation of hospital infection training

In the training satisfaction survey, the number of participants in the experimental group who were satisfied with the training’s ability to help with understanding and retention of knowledge, applying it in clinical practice, extrapolating knowledge, the appropriateness of the training methods, and the promotion of motivation and proactivity was significantly higher than in the control group (86 vs. 71, 82 vs. 62, 83 vs. 67, 88 vs. 73, 89 vs. 59). The results of the chi-square test for each satisfaction metric indicate that there were statistically significant differences between the control and experimental groups (all *p* < 0.05), as shown in Table 5.

**Table 5:** Analysis of the results of the satisfaction survey of the control and experimental groups.

Variables	Group		*x* ^2^	*P*
	Control group (*n* = 98)	Experimental group (*n* = 98)		
Contribute to the understanding and memorization of knowledge	71 (72.45)	86 (87.76)	7.202	0.007
Contributes to the application of knowledge in the clinical setting	62 (63.27)	82 (83.67)	10.470	0.001
Helps to learn by example	67 (68.37)	83 (84.69)	7.271	0.007
Reasonable training methods	73 (74.49)	88 (89.80)	7.826	0.005
Fully mobilize motivation and initiative	59 (60.20)	89 (90.82)	24.831	<0.001

## 4 Discussion

This study explored the effectiveness of the DOPS method in hospital infection control training by comparing an experimental group, which received both conventional training and formative assessments based on DOPS, with a control group which received only conventional training. Most participants were experienced medical personnel with over 10 years of work experience, indicating that even seasoned professionals can benefit from the DOPS method. Infection control skills are crucial for maintaining hospital safety and preventing Healthcare-associated infections, requiring continuous reinforcement and assessment, regardless of the level of professional experience (12, 13).

Our study demonstrated that the DOPS method introduced a structured, practical approach that emphasized direct observation and timely feedback during infection control procedures. This model enabled infection control staff to demonstrate their skills in real clinical scenarios while receiving immediate, constructive feedback from assessors (14). Unlike traditional training, which often focuses more on theory aspects or generalized practice, DOPS provides personalized, case-specific guidance, helping participants quickly identify and address their weaknesses (15). This process of real-time feedback and reassessment fostered more targeted and consistent improvements in procedural skills. During the study, the experimental group showed steady improvement in key infection control practices, such as the proper use of personal protective equipment and emergency management. The findings of our study align with previous research (16), which has similarly highlighted the effectiveness of DOPS in improving procedural skills and knowledge retention. DOPS would contributed to measurable improvements in hand hygiene practices and compliance rates among infection control personnel (17). Moreover, while our study underscores the dual benefits of DOPS in reinforcing both practical and theory competencies, similar findings have been reported in other healthcare training contexts. The use of DOPS in surgical training not only improved technical skills but also demonstrated better retention of surgical principles and theory knowledge (18).

According to the DOPS evaluation results, the DOPS method helped medical personnel strengthen their ability to respond effectively to medical situations through continuous assessment and feedback. This training model aligns well with the needs of infection control, where mastering technical skills is essential for the safety of both patients and medical personnel. Moreover, the study demonstrated that even after adjusting for various covariates such as age, gender, professional experience, and educational background, the experimental group not only excelled in practical infection control skills but also significantly outperformed the control group in theoretical knowledge assessments. Our research highlights the dual benefits of the DOPS method, while its primary focus is on improving practical procedural skills, it also has a profound impact on the acquisition and retention of theoretical knowledge (19). DOPS in reinforcing theoretical knowledge can likely be attributed to its unique approach of integrating theoretical concepts with real-world practice (20). Unlike traditional methods, which typically present theoretical knowledge in isolation (21), DOPS immerses participants in clinical scenarios where they must actively apply infection control principles (22). This practical learning experience not only makes abstract concepts more concrete but also deepens participants’ understanding by allowing them to observe how these principles are implemented in practice (23, 24). By directly engaging in infection control procedures, participants are better able to internalize the knowledge, making it easier to recall and apply in future clinical situations. Additionally, the continuous feedback provided through DOPS likely plays a crucial role in solidifying participants’ understanding. Personalized, real-time feedback allows participants to immediately address any misunderstandings or gaps in their knowledge, ensuring that theoretical concepts are accurately and effectively reinforced (25). This iterative process of assessment, feedback, and improvement enables participants to refine both their practical and theoretical skills in a structured and meaningful way, resulting in improved retention rates and a deeper, more enduring mastery of infection control principles (26). Our study also found no significant difference between the experimental and control groups in the use of protective equipment. This may be because this skill is a fundamental aspect of infection control, with both groups likely having a strong baseline competency. Future interventions could incorporate simulation-based scenarios or enhanced feedback to refine these critical but routine skills further.

The satisfaction results revealed that DOPS provided participants with valuable subjective benefits, showing that the interactive nature of the method was both beneficial and motivating (15). The opportunity to receive personalized, actionable feedback after each assessment likely boosted their confidence and sense of progress (27). In contrast, conventional training may not offer the same level of direct, personal engagement, which could explain the lower satisfaction in the control group. The interactive and real-time nature of DOPS assessments not only made the learning process more dynamic but also catered to the unique learning needs of each participant, making the training more relevant and impactful (28, 29). Our study showed that the DOPS method demonstrated significant potential in enhancing both practical skills and theoretical knowledge in hospital infection control, making it a valuable addition to conventional training programs. Its structured approach to skill development, combined with immediate feedback and continuous improvement, suggests that DOPS could play a key role in raising infection control standards and reducing Healthcare-associated infections in hospital settings.

In conclusion, the DOPS method appears to be an effective tool for improving the practical skills and theoretical knowledge of infection control staff. By providing continuous, targeted feedback, it helps bridge the gap between theory and practice. The DOPS method shows great promise for broader application in infection control training and other areas of clinical education. The positive outcomes in skill acquisition and participant satisfaction suggest that DOPS should be considered for wider implementation as part of infection control training programs. Further research could explore its long-term impact on infection control outcomes and patient safety in medical institutions.

This study introduces several innovative aspects. First and foremost, it is the first to explore the application of the DOPS method in hospital infection control training, offering new insights into how DOPS can significantly enhance participants’ knowledge acquisition and practical skill application in a real-world medical setting. By incorporating direct observation and immediate feedback, DOPS allows participants to better grasp and apply critical infection control protocols, filling a gap in conventional training methods. Secondly, this research stands out by including a variety of covariates and conducting subgroup analyses. This approach allows for a more nuanced and precise understanding of the relationship between the use of DOPS and its training outcomes, accounting for factors such as participants’ age, gender, professional background, and prior experience. By controlling for these variables, the study ensures that its results are both accurate and reliable, offering a clearer picture of how DOPS contributes to improvements in infection control training. Our study also has several limitations. One major limitation is the reliance on subjective evaluation indicators, which could introduce observer bias or human error. To address this, future studies should incorporate more objective performance metrics to assess participants’ actual operational differences after undergoing various training methods. For example, standardized assessments or measurable outcomes related to infection control practices could provide a more accurate evaluation of skill improvements. Additionally, the relatively small sample size in this study limits the generalizability of the findings. While the results indicate that DOPS has positive effects, expanding the sample size in future studies would help to stabilize the results and confirm the method’s broader applicability across different medical settings. A larger study population would also allow for more detailed subgroup analyses, further enriching our understanding of the impact of DOPS in various clinical contexts.

## 5 Conclusion

This study highlights the significant value of the DOPS method in enhancing hospital infection control training. By combining direct observation and real-time feedback with traditional training, DOPS not only improved practical infection control skills but also increased knowledge retention and participant satisfaction, making it a promising approach for broader implementation in infection control education.

## Data Availability

The raw data supporting the conclusions of this article will be made available by the authors, without undue reservation.
