# Supplementary material for: Application of directly observed procedural skills in hospital infection training: a randomized controlled trial
Source: Front Med (Lausanne). 2025 May 13;12:1509238. doi: 10.3389/fmed.2025.1509238 (PMC12106404; doi:10.3389/fmed.2025.1509238)
Supplement: Supplementary file 1 [file Data_Sheet_1.docx]

Supplementary Material

# Supplementary Figures and Tables

## Supplementary Figures


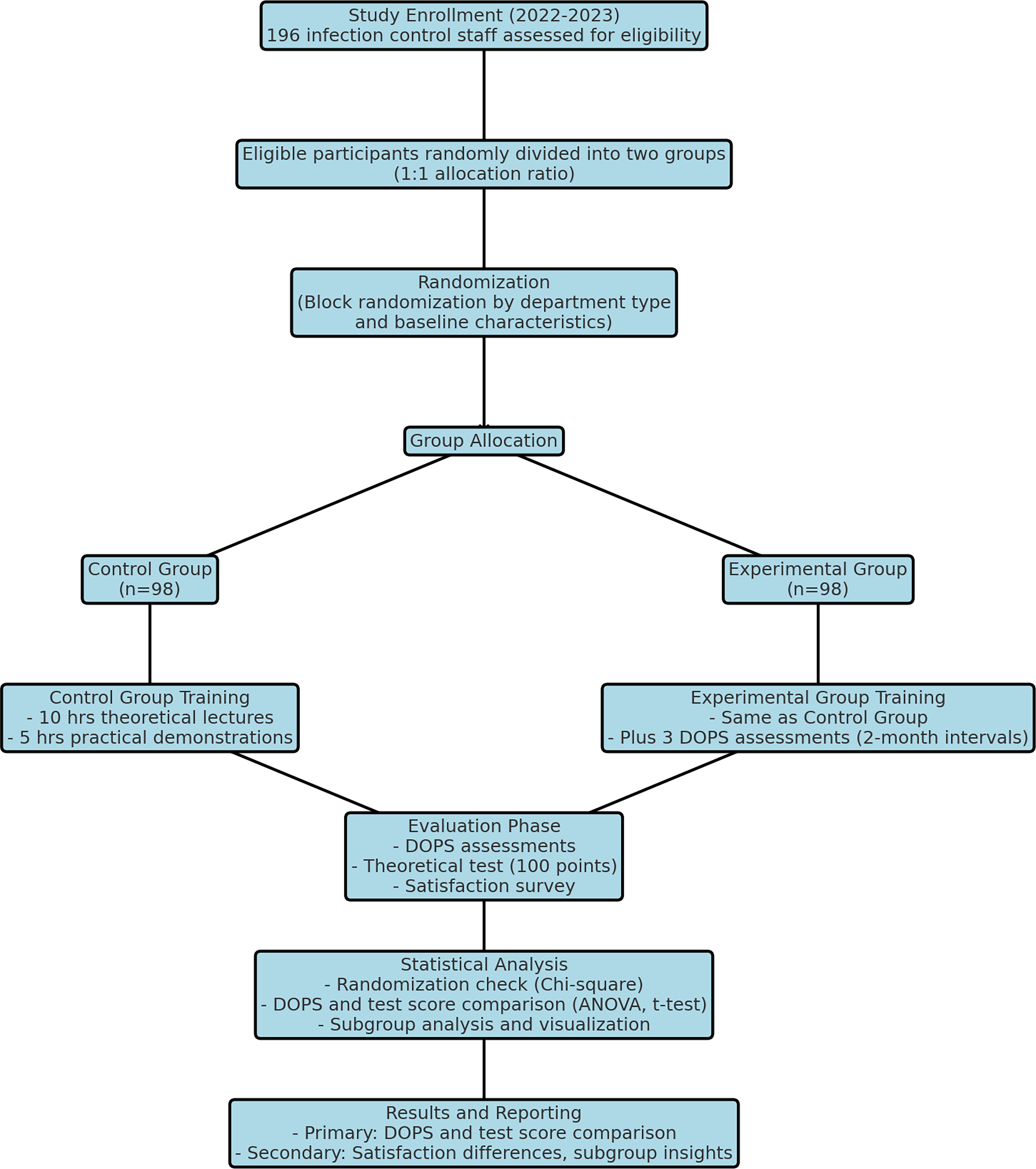


**Supplementary Figure 1.** The consort diagram of study methodology.


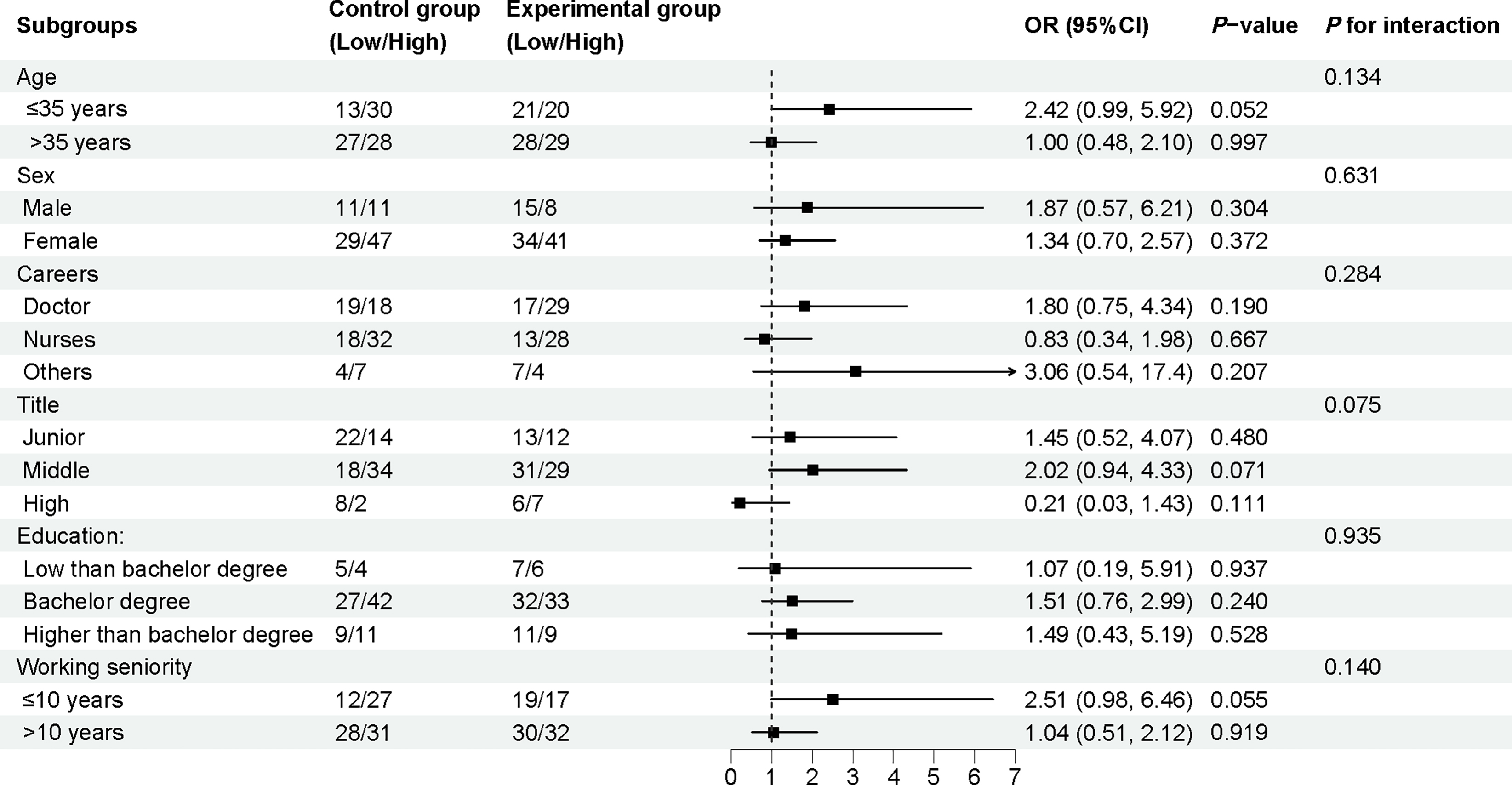


**Supplementary Figure 2.** Subgroup analysis of the odds ratio of the use of protective equipment comparing the control and experimental groups.

**
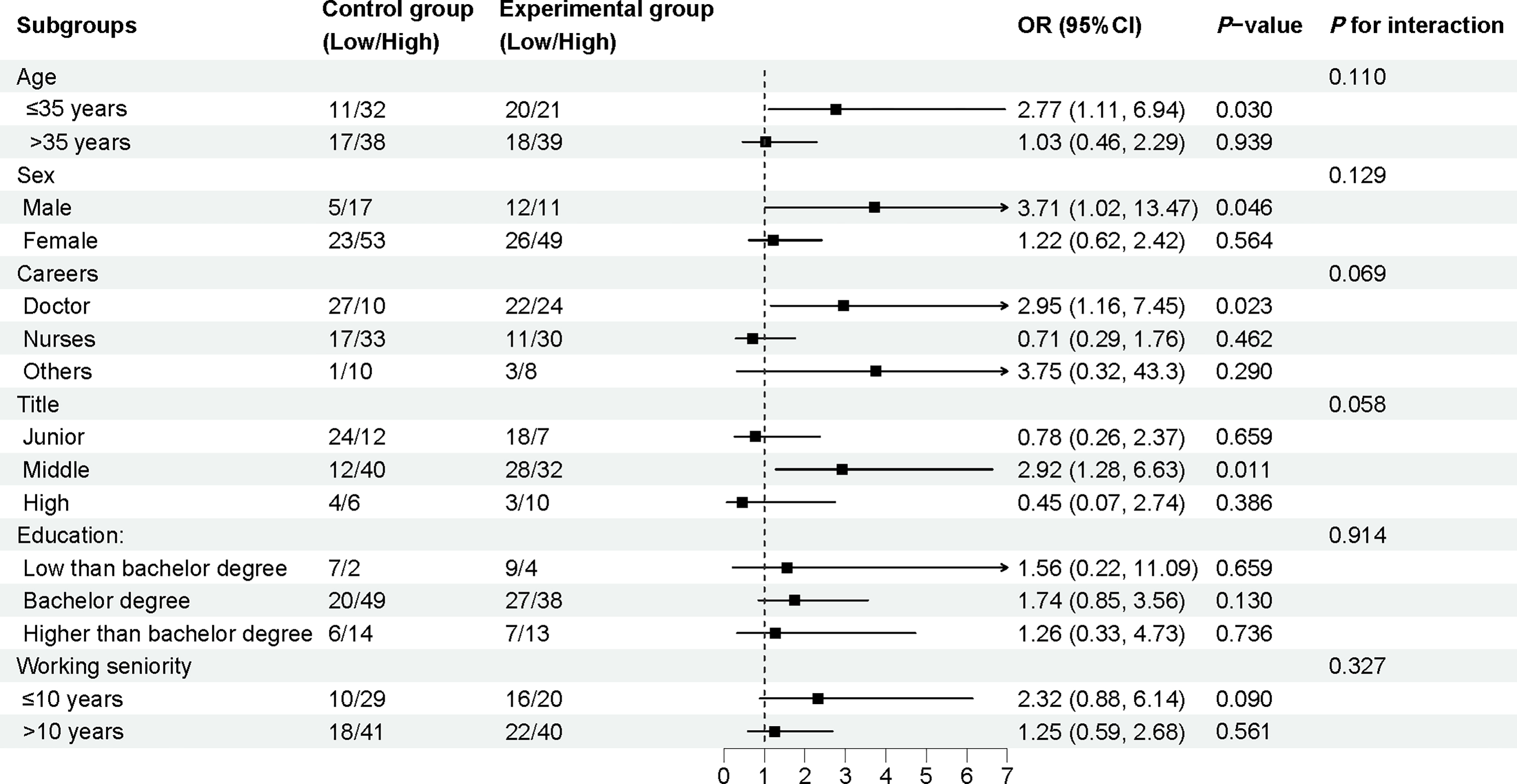
**

**Supplementary Figure 3.** Subgroup analysis of the odds ratio of hand hygiene norms comparing the control and experimental groups.


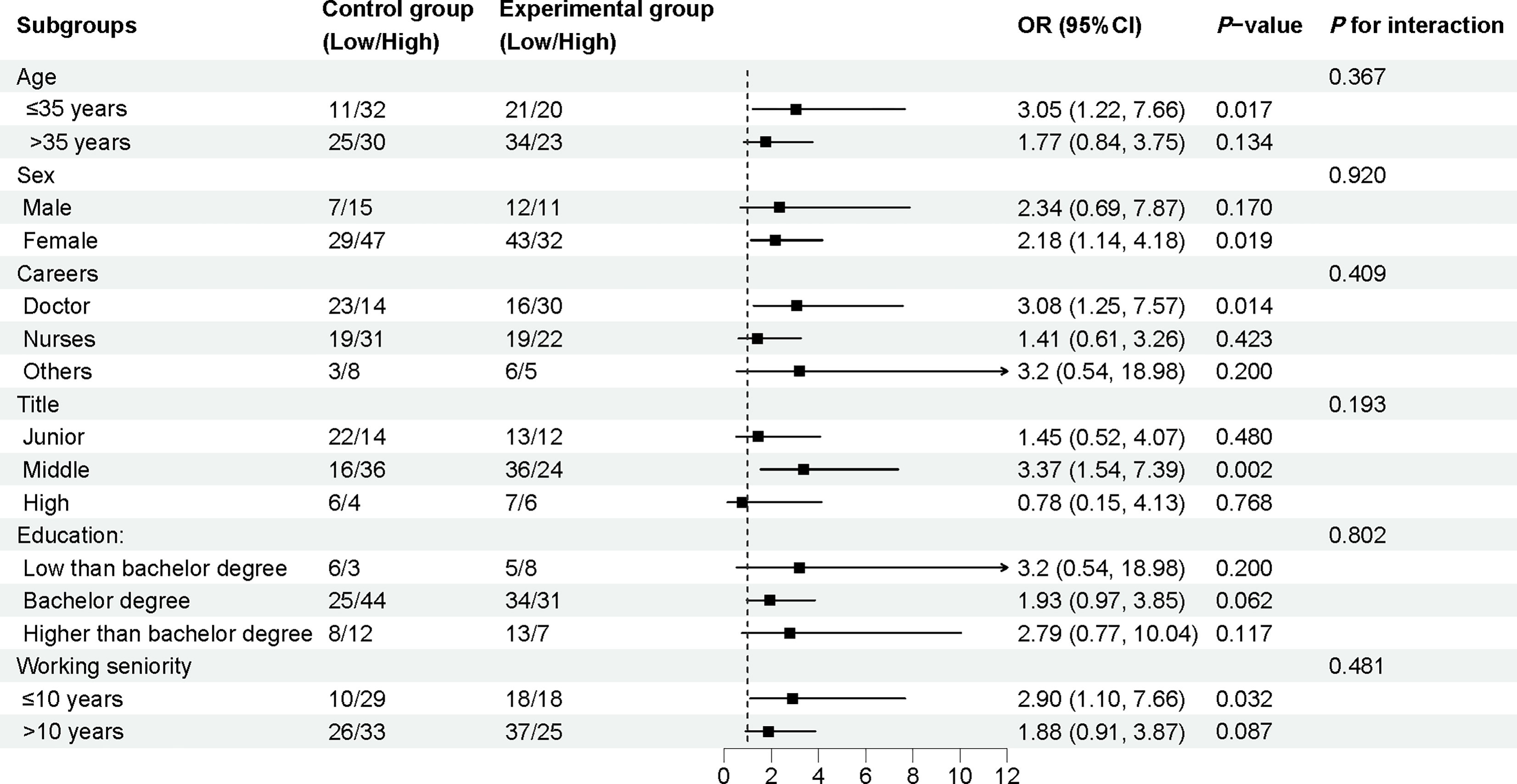


**Supplementary Figure 4.** Subgroup analysis of the odds ratio of aseptic techniques comparing the control and experimental groups.


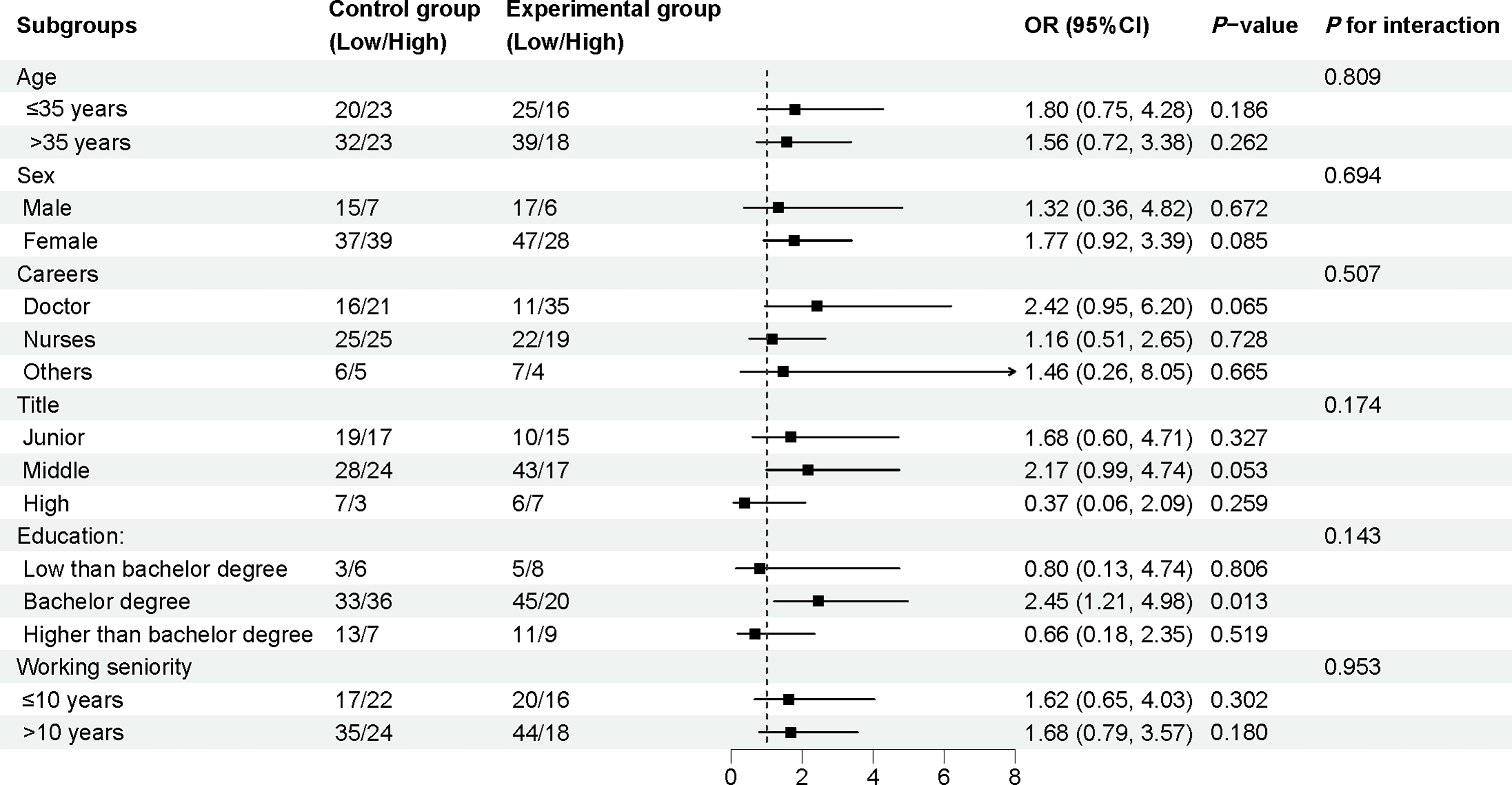


**Supplementary Figure 5.** Subgroup analysis of the odds ratio of prevention of needlestick injuries comparing the control and experimental groups.


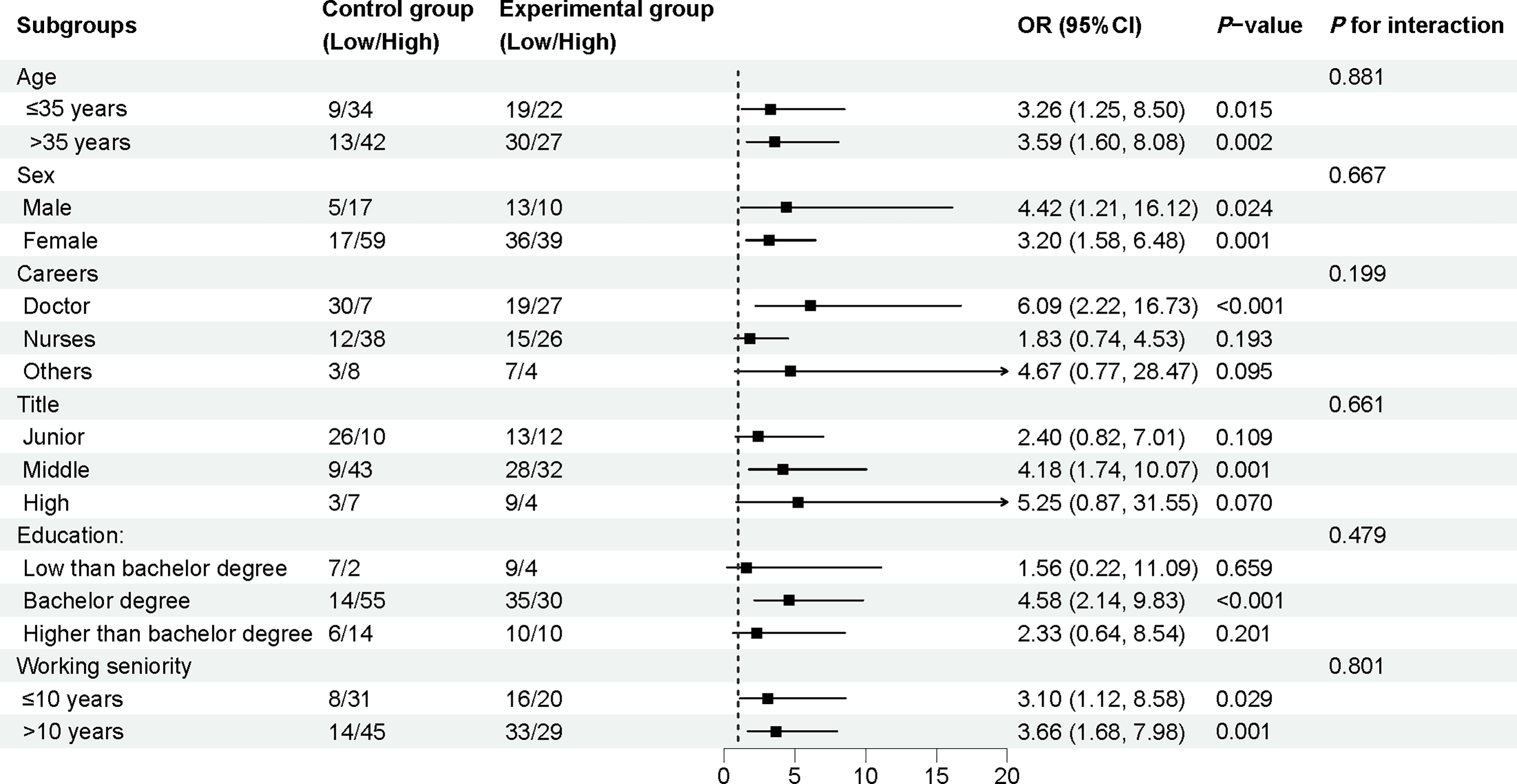


**Supplementary Figure 6.** Subgroup analysis of the odds ratio of medical waste classification comparing the control and experimental groups.
